# Hair Repigmentation Outcomes in Patients With Graying Hair Treated With Exosome Therapy: A Cross‐Sectional Observational Study

**DOI:** 10.1111/jocd.70526

**Published:** 2025-11-07

**Authors:** Suparuj Lueangarun, Patrick Po‐Han Huang, Wan‐Yi Chou, Elina Theodorakopoulou, Daniela Lemes

**Affiliations:** ^1^ Department of Aesthetic Medicine, College of Integrative Medicine Dhurakij Pundit University Bangkok Thailand; ^2^ Division of Dermatology DeMed Clinic Center Bangkok Thailand; ^3^ Huang PH Dermatology and Aesthetics Kaohsiung Taiwan (ROC); ^4^ Sincere Dermatology Clinic Taipei City Taiwan (ROC); ^5^ Pretty You Dermatology Clinic Athens Greece; ^6^ DL Dermatology and Laser Clinic Rio de Janeiro Brazil

**Keywords:** androgenetic alopecia, exosomes, gray hair, hair repigmentation, rose stem cell exosomes

## Abstract

**Background:**

Hair graying is an age‐associated condition primarily caused by the depletion and dysfunction of melanocyte stem cells within hair follicles. Emerging regenerative strategies, including exosome‐based therapies, offer the potential to restore pigmentation by targeting underlying cellular mechanisms.

**Objective:**

To investigate the clinical outcomes and correlates of hair repigmentation following exosome‐based therapy in individuals with gray hair.

**Methods:**

This cross‐sectional observational study enrolled 10 patients with visible gray or white hair who were treated with rose stem cell‐derived exosomes (RSCEs) using various procedures. Gray hair repigmentation outcomes were assessed using a standardized scale. Statistical correlations between clinical variables and treatment responses were analyzed.

**Results:**

A mean of 4.6 ± 1.3 treatment sessions was performed. Repigmentation was observed after an average of 2.4 ± 0.7 sessions and was maintained for 4.7 ± 1.9 months. The various treatment modalities also demonstrated efficacy in both hair regrowth and hair repigmentation. The mean outcome score was 2.8 ± 0.78, with 60% achieving a higher‐grade response (≥ 50% improvement). Shorter duration of graying (*p* = 0.0363), presence of androgenetic alopecia (AGA) (*p* = 0.0332), and moderate baseline severity (Stage 2) (*p* = 0.0133) were significantly associated with better outcomes. No adverse events were reported.

**Conclusion:**

Exosome‐based therapy appears to be a safe and promising approach for inducing hair repigmentation in individuals with gray hair using various treatment modalities. Factors such as the presence of AGA, shorter duration of graying, and moderate baseline severity may predict a better response. Larger, controlled studies are warranted to validate these findings and help standardize treatment protocols.

## 1 Introduction

Hair graying is a common, age‐associated process primarily driven by the depletion, dysfunction, and migration failure of melanocyte stem cells (MeSCs) within the hair follicle niche [1]. These changes are exacerbated by intrinsic and extrinsic factors such as oxidative stress [2], mitochondrial DNA damage, inflammation [3], and niche aging, leading to an irreversible loss of pigment production in the hair shaft [4]. Although cosmetic dyes offer temporary coverage, they do not address the underlying pathophysiology and may cause allergic reactions or scalp irritation with repeated use [5].

Recently, exosome‐based therapy has emerged as a novel regenerative approach targeting the cellular and molecular causes of graying. Exosomes are nano‐sized extracellular vesicles secreted by mesenchymal stem cells (MSCs) and other progenitor cells, carrying microRNAs, mRNAs, proteins, and signaling lipids that influence target cells through paracrine signaling [6]. Preclinical studies have demonstrated that MSC‐derived exosomes can delay hair follicle regression and promote melanogenesis by activating key signaling pathways such as Wnt/β‐catenin, all of which are crucial for MeSCs' survival and function [7]. Additionally, exosomes exhibit anti‐inflammatory [8] and antioxidative properties [2], potentially restoring the oxidative balance in the hair follicle microenvironment and protecting melanogenic cells from further damage [2]. Moreover, a prior case report showed successful repigmentation in poliosis circumscripta using exosomes combined with fractional picosecond laser [9, 10]. However, clinical data on their application for treating hair graying remain limited.

This cross‐sectional study aims to investigate the effects and correlations of exosome‐based treatment for hair repigmentation in individuals with hair graying, providing preliminary evidence of its therapeutic potential in reversing or reducing visible graying.

## 2 Research Methodology

The cross‐sectional observational study was designed to evaluate the outcomes and correlation of exosome‐based therapy on hair repigmentation in patients presenting with graying hair. A total of 10 patients were enrolled. All participants had visible gray or white hair and were not undergoing any concurrent hair repigmentation treatments. Inclusion criteria required participants to be aged 18 years or older and treated with exosomes (Rose stem cell exosomes [RSCEs], ASCEplus HRLV; ExoCoBio Inc., Seoul, Korea), while individuals with autoimmune alopecia, active scalp conditions, or recent hair dye use were excluded. Baseline data were collected using a standardized case record form, which included demographic details (age, gender, ethnicity, country), Fitzpatrick skin phototype, underlying medical and dermatological conditions, history of androgenetic alopecia (AGA) (including Norwood stage and duration), as well as gray hair characteristics such as severity, duration, and family history. The treatment procedures were also recorded, including treatment methods, exosome dilution, treatment intervals, and clinical follow‐up after the final session. Currently, the majority of exosome‐based products are regulated as cosmetic formulations. Nonetheless, regulatory classifications differ across countries; for instance, in Thailand, specific exosome preparations such as ASCEplus (ExoCoBio Inc., Seoul, Korea) have been formally registered as medical devices [9].

Due to the lack of a standardized scale to determine hair graying severity and treatment response in the current literature, we employed the hair graying scale [11]—a standardized percentage‐based gray hair grading system—evaluated by two blinded dermatologists. This grading system classifies graying into five categories based on the estimated percentage of gray hair present. Grade 0 indicates no visible graying (0%), while Grade 1 corresponds to mild graying involving 1%–25% of the scalp hair. Moderate graying, defined as 26%–50% involvement, is categorized as Grade 2. Grade 3 reflects severe graying with 51%–75% of hair affected, and Grade 4 represents very severe or complete graying, with more than 75% up to 100% gray hair.

Hair repigmentation was assessed based on a hair repigmentation scale, graded by two blinded dermatologists and modified from the hair graying scale [11]: 0 (no change), 1 (0%–25% improvement), 2 (25%–50% improvement), 3 (50%–75% improvement), and 4 (75%–100% improvement). The hair repigmentation scale was classified into two categories: mild response (score 1–2) and higher response (score 3–4).

Androgenetic alopecia treatment outcomes were evaluated using global photographs taken with a digital camera. The images were clinically assessed by two blinded dermatologists using a standardized seven‐point rating scale (−3 = greatly decreased, −2 = moderately decreased, −1 = slightly decreased, 0 = no change, +1 = slightly increased, +2 = moderately increased, and +3 = greatly increased) [12, 13].

This observational case series was conducted in accordance with the Declaration of Helsinki and Good Clinical Practice. All patients provided written informed consent for treatment and anonymized image use.

### 2.1 Statistical Analysis

Statistical analysis was performed to evaluate the correlation between clinical variables and gray hair repigmentation outcomes following exosome‐based therapy. Descriptive statistics were used to summarize baseline demographics, treatment characteristics, and outcomes. Continuous variables were expressed as mean ± standard deviation (SD), while categorical variables were reported as counts and percentages. Comparisons of repigmentation scores between two independent groups (e.g., gender, ethnicity, AGA status) were analyzed using the Mann–Whitney U test. Categorical outcome comparisons, particularly in groups with small sample sizes, were assessed using Fisher's exact test. Spearman's rank correlation coefficient (*r*) was employed to evaluate associations between ordinal or continuous variables—such as the duration of graying, AGA severity, or number of treatment sessions—and repigmentation outcomes. Treatment efficacy was graded on a standardized 4‐point scale, with scores of 3–4 indicating a higher response (≥ 50% improvement), and scores of 1–2 considered a mild response. A *p* value of less than 0.05 was considered statistically significant. All statistical analyses were conducted using STATA/MP version 18.

## 3 Results (Table 1, Figures 1, 2, 3, 4, 5, 6, 7, 8, 9, 10)

### 3.1 Baseline Characteristics

Ten patients (6 males, 4 females; mean age 58.6 ± 10.2 years) with visible gray or white hair were enrolled. Most were of Asian ethnicity (70%) and had Fitzpatrick skin types II–III (70%). AGA was present in 70%, with a mean duration of 12.3 ± 6.8 years and Norwood stages III–VI. The mean duration of gray hair was 13.1 ± 5.7 years, and 70% reported a family history of hair graying. Baseline gray hair severity was Grade 1 in 10%, Grade 2 in 50%, Grade 3 in 20%, and Grade 4 in 20%.

**TABLE 1:** Summary of patient characteristics and treatment outcomes.

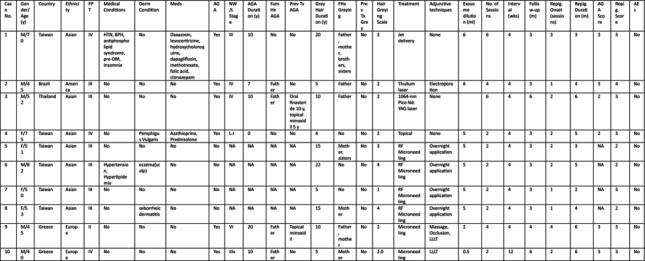

Abbreviations: 1064‐nm pico Nd:YAG laser, Fractional 1064‐nm Nd:YAG picosecond laser; AEs, adverse events/side effects; AGA, androgenetic alopecia; Derm condition, dermatologic condition; Exosome dilution (mL), exosomes 1 vial diluted in (mL); F, female; Fam Hx AGA, family history of androgenetic alopecia; FHx graying, family history of hair graying; Follow‐up (m), duration of follow‐up from the last treatment (m); FPT, Fitzpatrick skin phototype; Interval (wks), interval between each treatment (wks); Jet delivery, Jet‐based transdermal delivery device; L, Ludwig classification for female‐pattern hair loss; LLLT, red low level laser therapy; M, male; m, months; Meds, current medication; mL, milliliters; NA, data not available; NW, Norwood‐Hamilton classification for male‐pattern hair loss; Overnight application, exosomes left on the skin overnight for three consecutive nights without rinsing; Prev Tx AGA, previous treatment of AGA; Prev Tx gray, previous treatments for gray hair; Repig. duration (m), duration of visible repigmentation from the last treatment; Repig. onset (sessions), repigmentation first seen (sessions); RF microneedling, fractional RF microneedling; Thulium laser, fractional 1927 nm thulium laser; Topical, topical application; wk, week (s); y, years.

**FIGURE 1:**
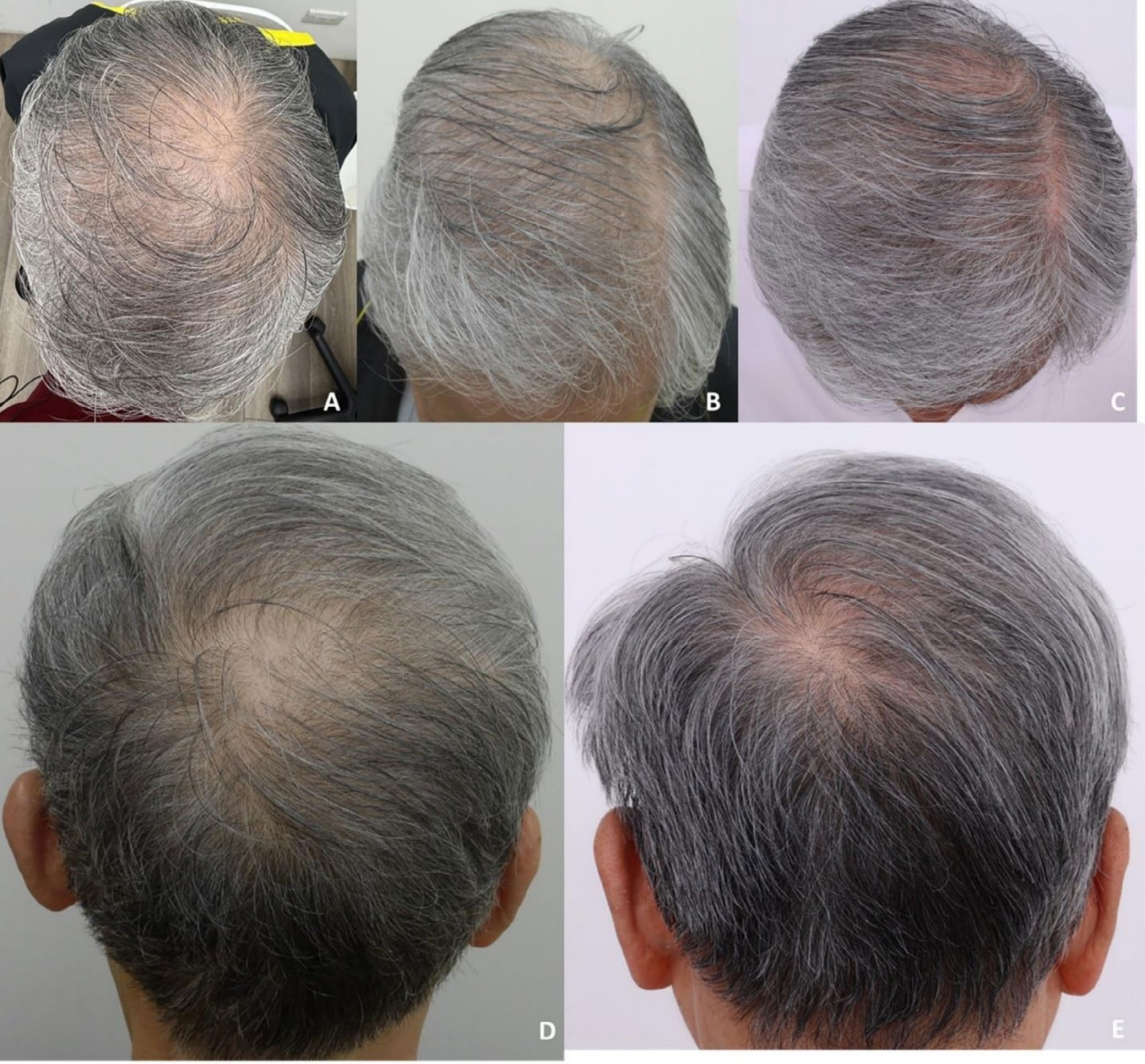
Case 1: Hair repigmentation following six sessions of exosome treatment using a jet‐based transdermal delivery device. Vertex views at baseline (A), 4 weeks after three treatment sessions (B), and 12 weeks after six treatment sessions (C) demonstrate progressive repigmentation of gray hair. Occipital views at 4 weeks after three sessions (D) and 12 weeks after six sessions (E) show increased pigment density and darkening of hair shafts.

**FIGURE 2:**
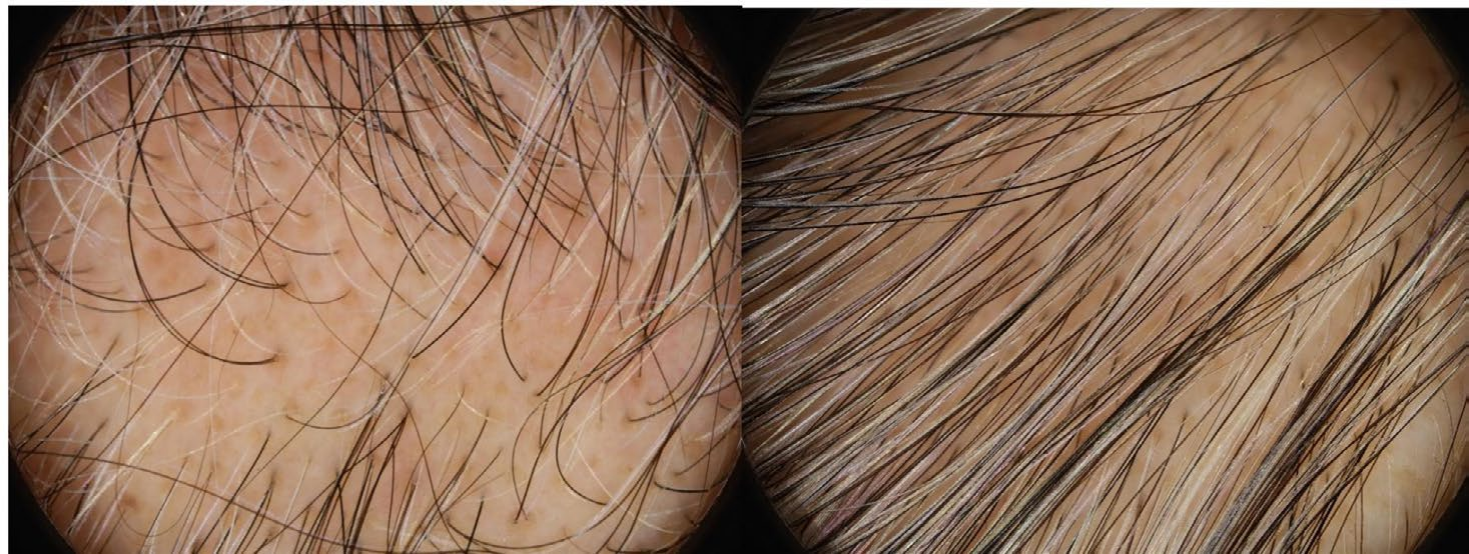
Case 1: Close‐up images demonstrate a noticeable color shift from whitish to yellowish hair shafts after six treatment sessions (8× magnification, captured using a Casio DZ‐D100 digital camera).

**FIGURE 3:**
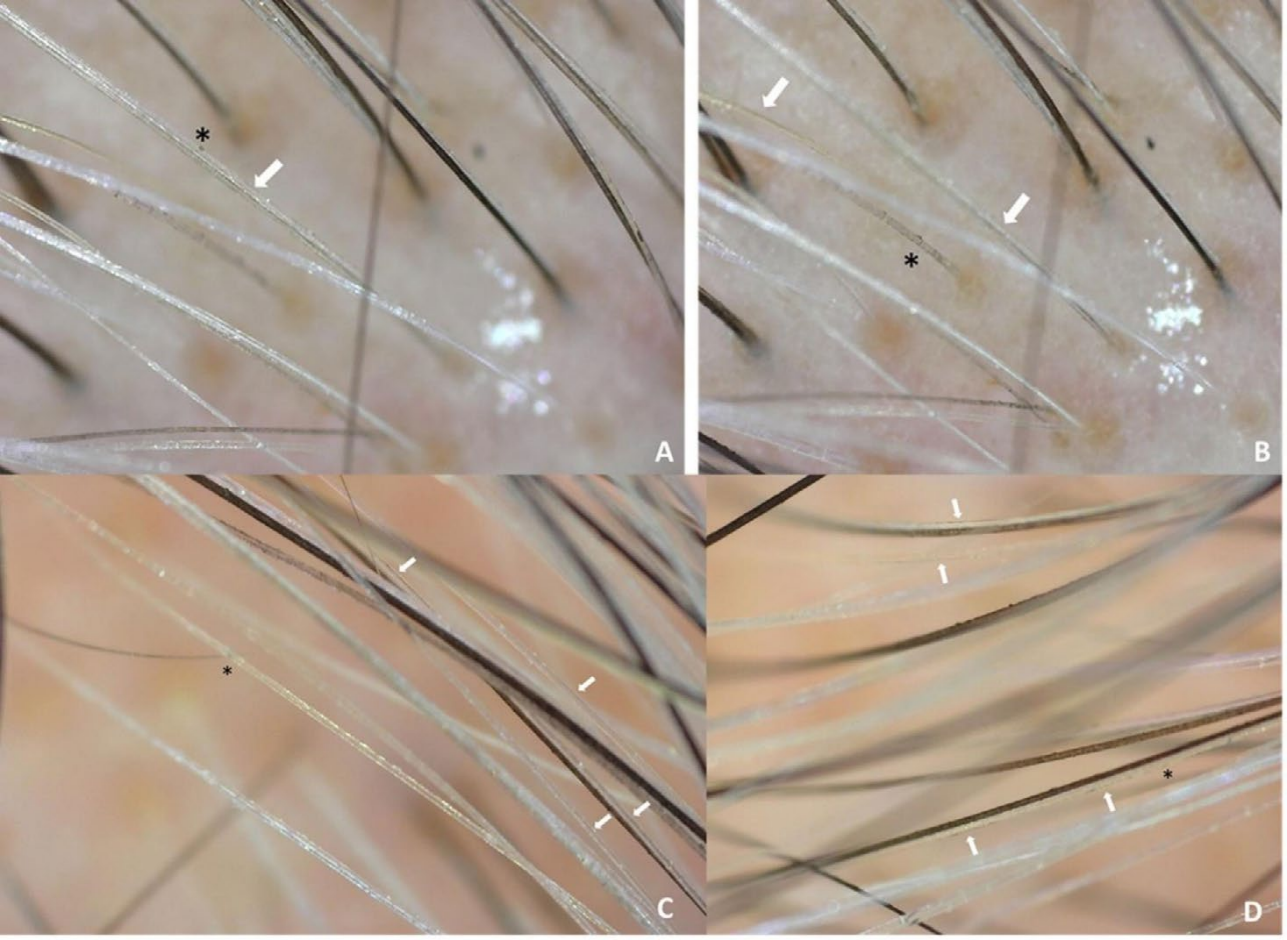
Case 1 (A–D): Trichoscopic images after six treatment sessions showing progressive hair shaft pigmentation. Microscopic views (200× magnification) of the vertex area illustrate the transition of hair shafts from whitish to yellowish. Early repigmentation is evident with scattered blackish granules (*) and linear pigmentation (arrow) within the yellowish shafts (Dino‐Lite Digital Microscope, AnMo Electronics Corporation, Taiwan).

**FIGURE 4:**
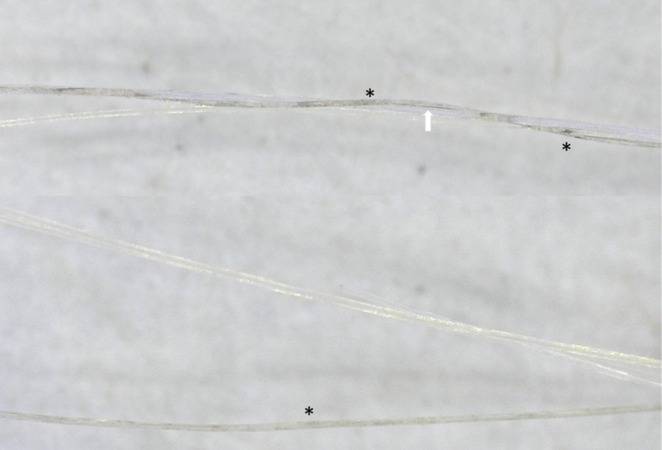
Case 1: Trichoscopic images (200× magnification) show whitish hair shafts transitioning to yellowish coloration. Early repigmentation is evident with blackish granules (*) and linear pigmentation (arrow) within the shaft structure, suggesting partial melanogenesis (Dino‐Lite Digital Microscope, AnMo Electronics Corporation, Taiwan).

**FIGURE 5:**
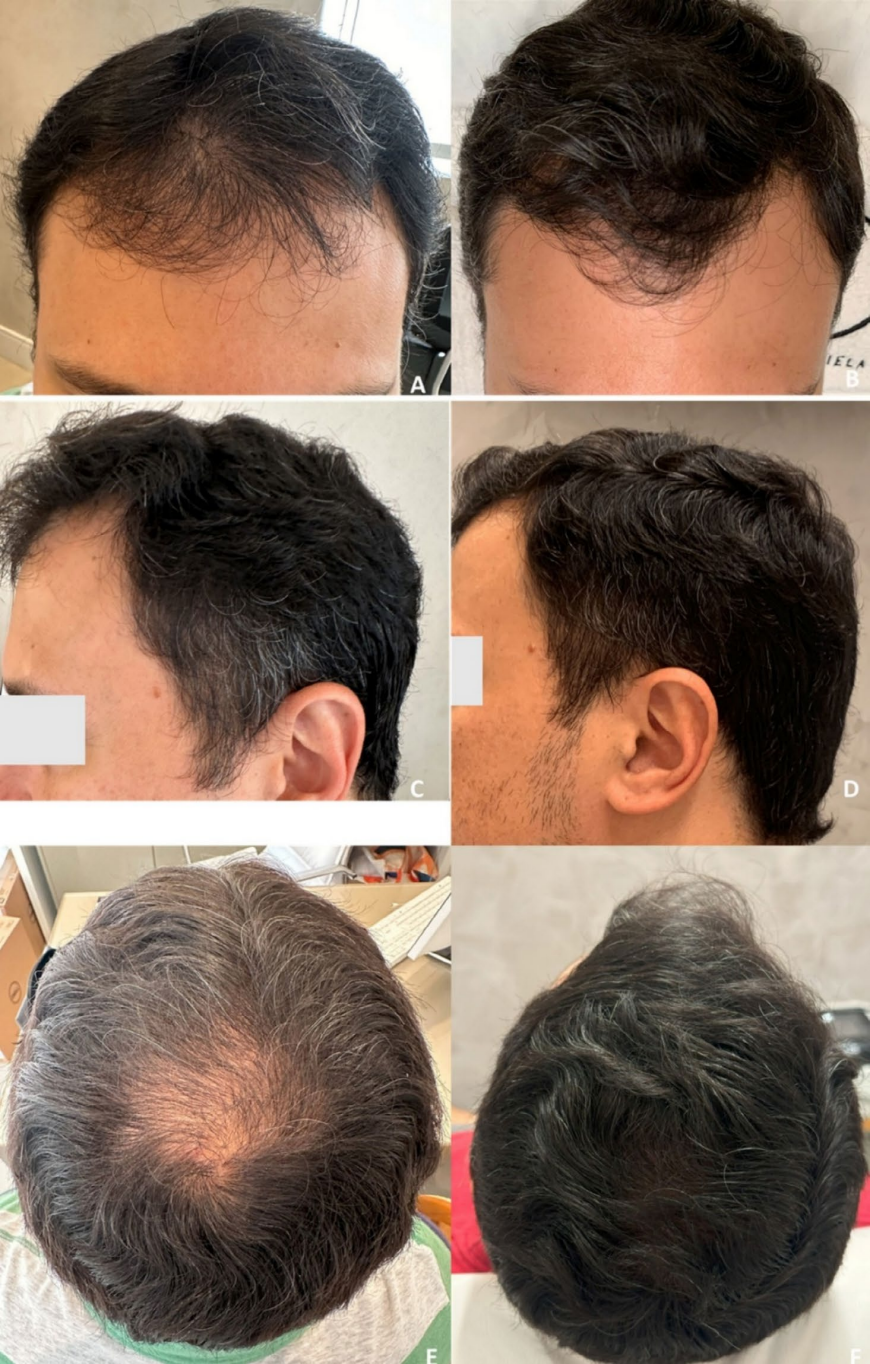
Case 2: Hair repigmentation following fractional 1927 nm thulium laser treatment combined with topical exosomes. Frontal views at baseline (A) and after 16 weeks of four sessions at 4‐week intervals (B) show increased darkening and density of the frontal hairline. Left lateral views before (C) and after treatment (D) demonstrate significant pigment restoration of temporal gray hair. Vertex views at baseline (E) and after treatment (F) reveal visible darkening of gray hair and improved coverage.

**FIGURE 6:**
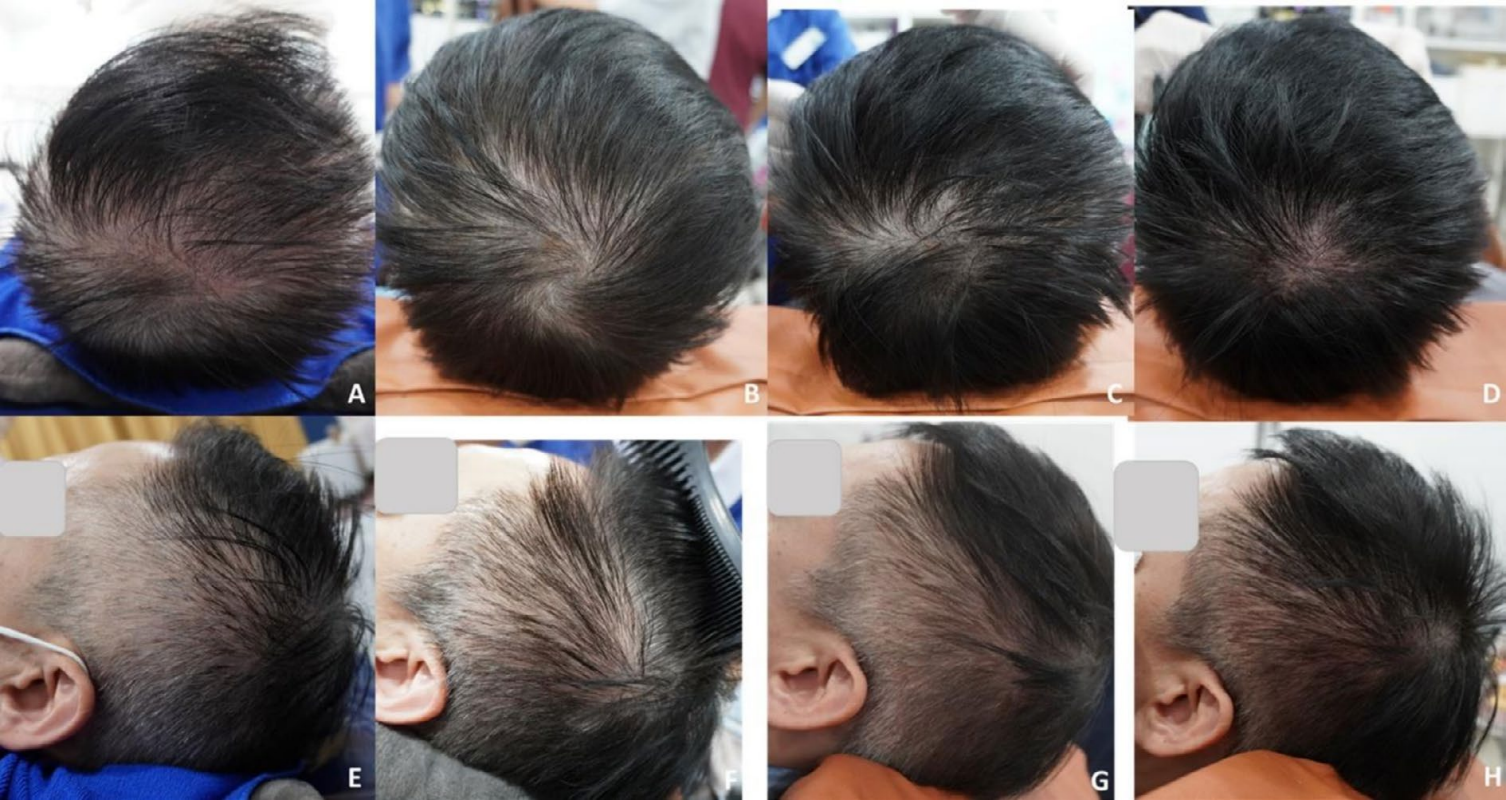
Case 3: Hair repigmentation after six treatment sessions at 4‐week intervals using fractional 1064‐nm Nd: YAG picosecond laser combined with electroporated exosome delivery. Vertex views at baseline (A), 4 weeks after two sessions (B), 4 weeks after six sessions (C), and 24 weeks after completing six sessions (D) show progressive darkening of hair shafts. Lateral views of the left scalp at baseline (E), 4 weeks after two sessions (F), after six sessions (G), and 24 weeks post‐treatment (H) demonstrate visible pigment restoration and hair regrowth.

**FIGURE 7:**
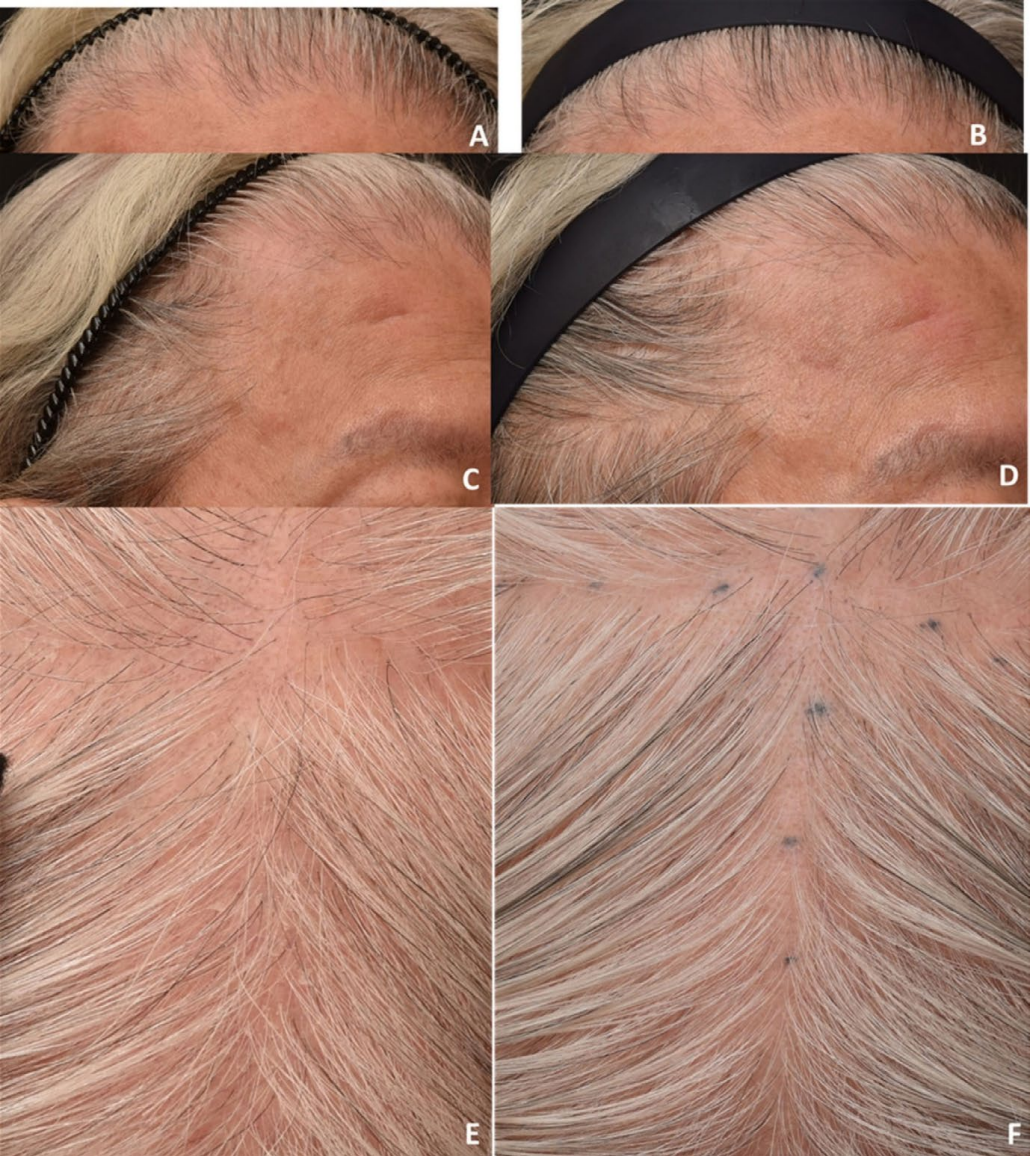
Case 6: Hair repigmentation after two sessions of fractional radiofrequency microneedle combined with topical exosome application. Frontal views at baseline (A) and 12 weeks after two sessions (B). Left lateral views at baseline (C) and 12 weeks after treatment (D). Vertex views at baseline (E) and 12 weeks post‐treatment (F) demonstrate repigmentation of depigmented hair shafts.

**FIGURE 8:**
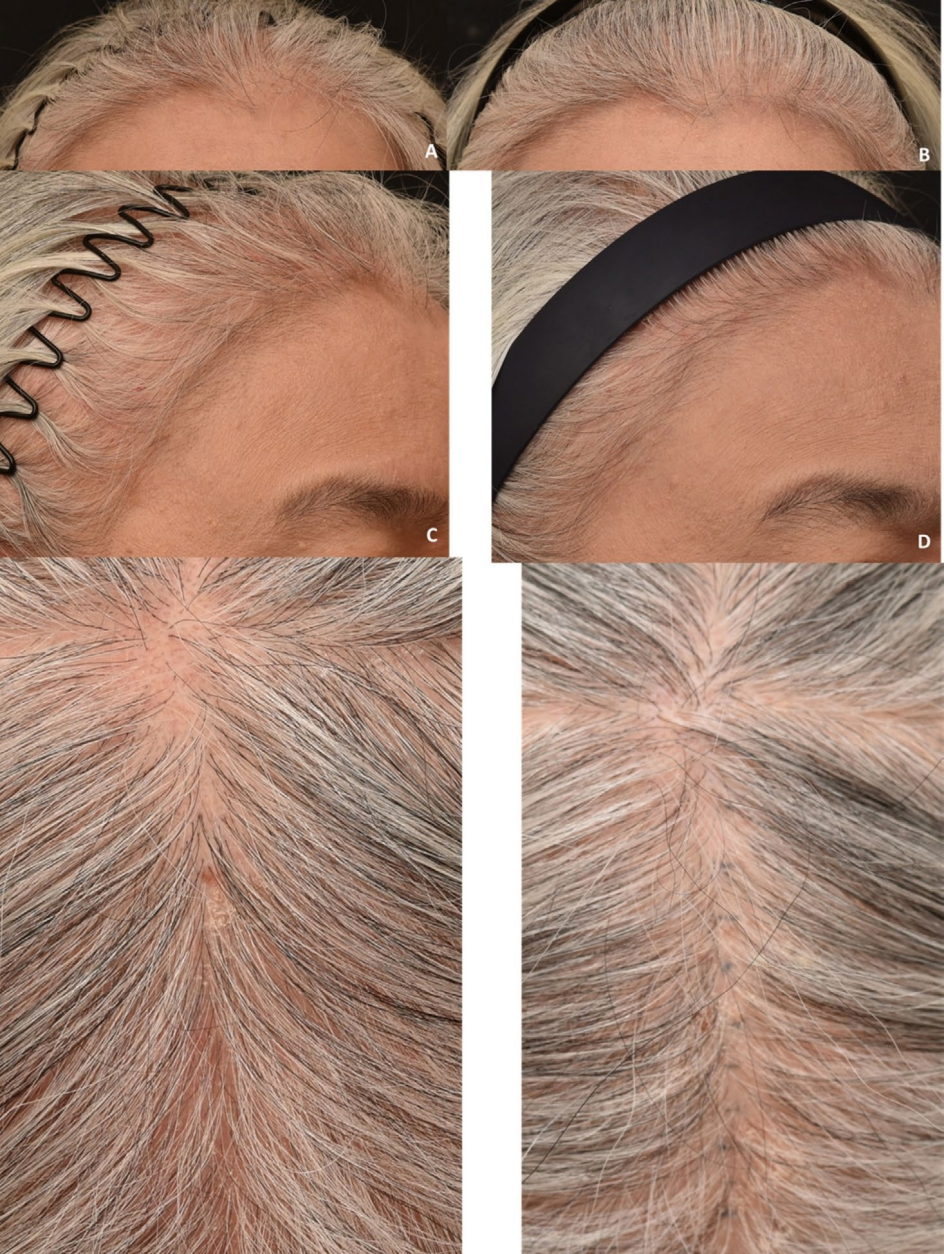
Case 8: Hair repigmentation after two sessions of fractional radiofrequency microneedle combined with exosome application. Frontal views at baseline (A) and 12 weeks after treatment (B). Left lateral views at baseline (C) and 12 weeks (D). Vertex views show scattered pigmented hair shafts at baseline (E) and more prominent repigmentation at 12 weeks post‐treatment (F).

**FIGURE 9:**
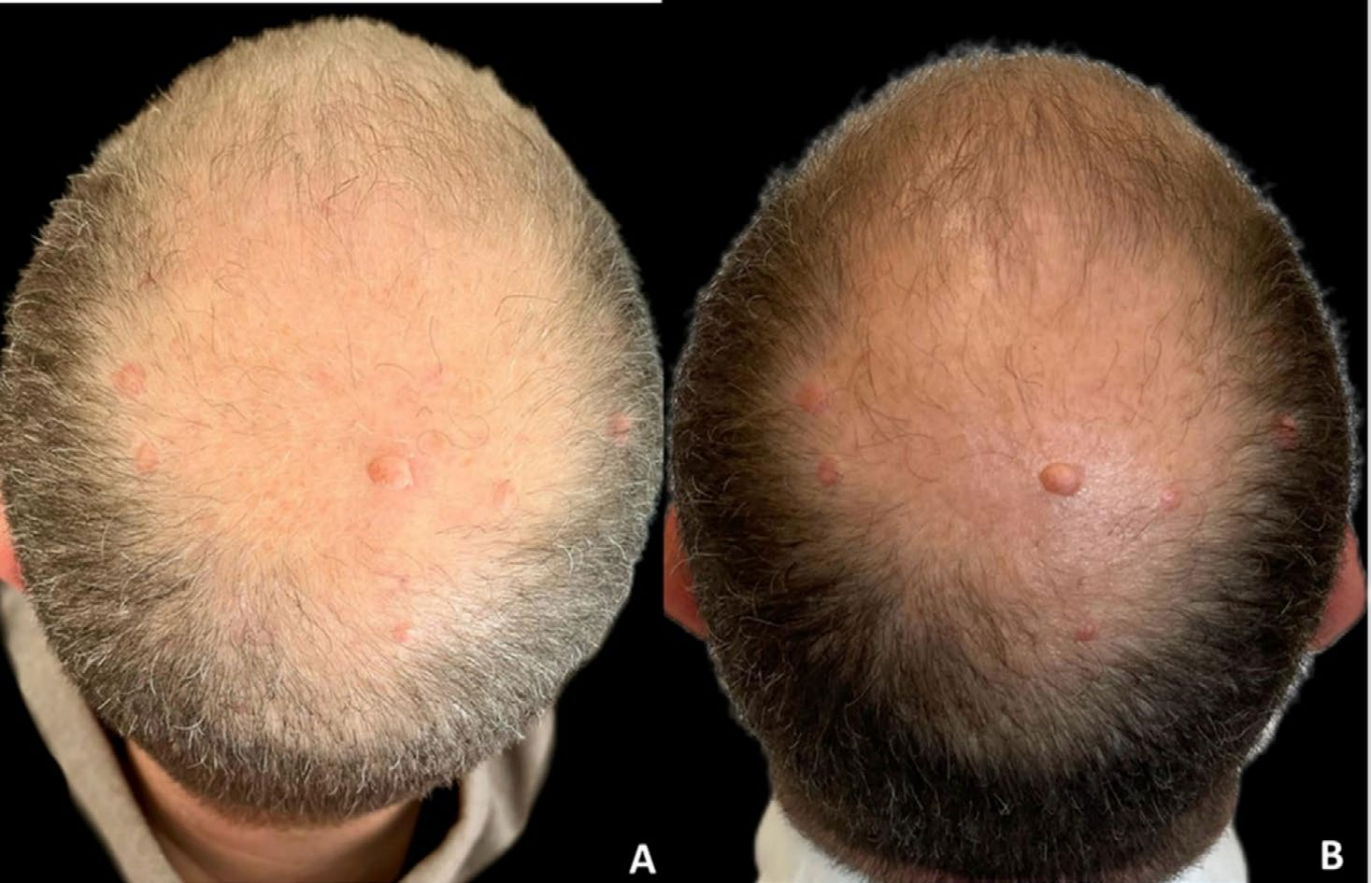
Case 9: Hair repigmentation after four sessions of microneedling combined with exosome application. Vertex views at baseline (A) and 16 weeks after treatment (B). Marked improvement in hair pigmentation and density is observed post‐treatment.

**FIGURE 10:**
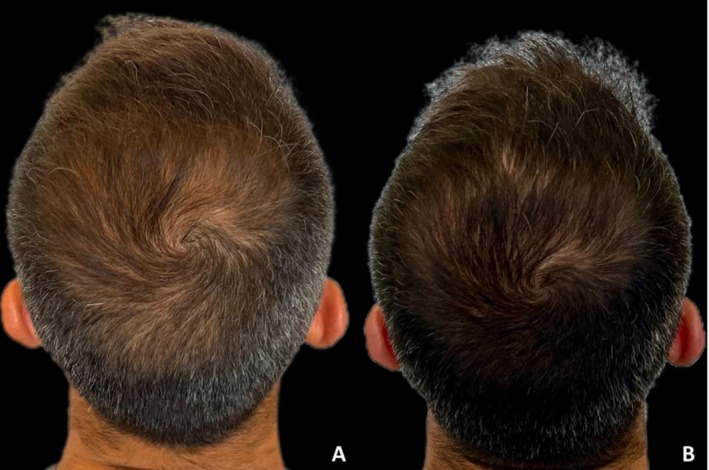
Case 10: Hair repigmentation after two sessions of microneedling combined with topical exosome application. Vertex views at baseline (A) and 24 weeks after the second session (B) demonstrate a marked increase in hair shaft pigmentation.

#### 3.1.1 Treatment Methods

Patients received an average of 4.6 ± 1.3 treatment sessions (range: 3–6) at 4‐week intervals. Exosomes were delivered using various methods most commonly fractional radiofrequency (RF) microneedling (40%), followed by microneedling (20%), jet‐based transdermal delivery device (10%), and laser‐assisted techniques (20%), including fractional 1927‐nm thulium and 1064‐nm picosecond Nd:YAG lasers [14]. One patient (10%) received topical application only. Each session involved the dilution of one vial of exosomes in normal saline, with a mean dilution volume of 4.45 ± 1.77 mL. The most common dilution was 5.0 mL (60%), and in one case (Case 10), two vials were diluted in 1.0 mL, resulting in a markedly higher concentration per unit volume.

#### 3.1.2 Treatment Outcomes

Patients were followed for a mean duration of 5.4 ± 1.8 months after their final treatment session. The earliest visible signs of hair repigmentation were observed, on average, after 2.4 ± 0.7 sessions. Once repigmentation was achieved, the visible effect was sustained for an average of 4.7 ± 1.9 months from the last session. At the end of treatment, the mean gray hair repigmentation score was 2.8 ± 0.78 (4‐point scale). A higher‐grade response (score 3–4) was seen in 60% of patients, while 40% showed a mild response (score 1–2). No adverse effects were reported throughout the study. The median AGA score increased from 0 (0–0) at baseline to 3 [2, 3] after treatment, with a significant improvement (*p* = 0.031).

### 3.2 The Correlation of Clinical Variables With Gray Hair Repigmentation Outcomes Following Treatment (Table 2)

**TABLE 2:** Correlation of demographic and clinical variables with gray hair repigmentation outcomes following treatment.

Variables	Hair graying scale	*p*	Hair repigmentation scale		*p*
			Mild response (1, 2)	Higher response (3, 4)	
Gender		0.495^a^			0.500^b^
Male	3 ± 0.63		1 (33.33)	5 (71.43)	
Female	2.5 ± 0.58		2 (66.67)	2 (28.57)	
Age		0.150^c^			1.000^b^
< 60 years	*r* = −0.49		2 (66.67)	5 (71.43)	
≥ 60 years			1 (33.33)	2 (28.57)	
Ethnicity		0.250^a^			0.475^b^
Asian	2.57 ± 0.53		3 (100.0)	4 (57.14)	
Non‐Asian	3.33 ± 0.58		0 (0.0)	3 (42.86)	
Skin phototype		0.661^c^			0.475^b^
II–III	*r* = 0.158		3 (100.0)	4 (57.14)	
IV			0 (0.0)	3 (42.86)	
AGA		0.057^a^			**0.033** ^**b**^
Yes	3.17 ± 0.41		0 (0.0)	6 (85.71)	
No	2.25 ± 0.50		3 (100.0)	1 (14.29)	
Norwood stage		0.764^c^			—
III	*r* = 0.19			2 (40.00)	
IV				2 (40.00)	
VI				1 (20.00)	
Duration of AGA (years)		0.111^c^			—
7	*r* = −0.79			1 (20.00)	
10				3 (60.00)	
20				1 (20.00)	
Gray hair duration (years)	*r* = −0.66	**0.036** ^**c**^	17.33 ± 4.04	8.43 ± 5.68	**0.050** ^**a**^
Family history of hair graying		1.000^a^			1.000^b^
Yes	2.85 ± 0.69		2 (66.67)	5 (71.43)	
No	2.67 ± 0.58		1 (33.33)	2 (28.57)	
Gray hair severity stage		**0.013** ^**c**^			**0.050** ^**b**^
1	*r* = −0.75		0 (0.0)	1 (14.29)	
2			0 (0.0)	5 (71.43)	
3			1 (33.33)	1 (14.29)	
4			2 (66.67)	0 (0.0)	
AGA treatment outcome		0.402^c^			—
2	*r* = 0.42			2 (33.33)	
3				5 (66.67)	
Number of treatment sessions		0.111^c^			0.300^b^
2	*r* = 0.54		3 (100.0)	3 (42.86)	
4			0 (0.0)	2 (28.57)	
6			0 (0.0)	2 (28.57)	

*Note:* The bold values means statistically significant (*p* value < 0.05).

^a^ Mann–Whitney *U* test.

^b^ Fisher's exact test.

^c^ Spearman's correlation.

This analysis examined the correlation between clinical variables and gray hair repigmentation outcomes following exosome therapy. Gender, age, ethnicity, and skin phototype showed no significant impact, though males, younger patients, non‐Asians, and those with skin types II–III tended to have better outcomes. A significant correlation was observed with AGA, where affected patients had higher response rates (85.71%) and mean scores (3.17, *p* = 0.0332). Shorter gray hair duration (8.43 vs. 17.33 years, *p* = 0.0363) and moderate baseline severity (Stage 2) (*p* = 0.0133) were also associated with better outcomes. Other factors, including Norwood stage and treatment frequency, showed trends but were not statistically significant. These findings suggest that AGA presence, shorter duration of graying, and moderate severity may predict better treatment response.

## 4 Discussion

This cross‐sectional observational study offers clinical evidence and correlation supporting the efficacy of exosome‐based therapy in stimulating hair repigmentation in individuals with gray hair. A majority of participants (60%) demonstrated moderate to marked improvement, with a mean repigmentation outcome score of 2.8 ± 0.78 on a standardized scale. Interestingly, repigmentation was observed as early as the second to third treatment session in most patients and sustained for several months post‐treatment, suggesting sustained biological effects. However, not all patients achieved full repigmentation, and inter‐individual variability in response may be attributed to differences in baseline follicular melanocyte reserves, age, treatment delivery methods, or adjunctive therapies such as red low‐level laser therapy (LLLT) or electroporation. These findings contribute to the growing body of literature suggesting that extracellular vesicles derived from stem cells hold regenerative potential for pigmentary disorders [15], including hair graying.

Treatment response appeared to be influenced by several patient‐ and procedure‐related variables. Notably, patients with AGA showed significantly higher response rates (85.71%) and greater mean outcome scores (3.17 ± 0.41; *p* = 0.0332) than those without AGA. MeSCs are still present in AGA, but their regenerative function and pigment production are hindered [1, 16], primarily due to androgen‐induced reduction of stem cell factor (SCF) from dermal papilla cells [17]. This leads to impaired melanocyte activation and contributes to the hair graying observed in AGA. Exosome therapies that restore signaling and improve the scalp microenvironment may help reactivate these MeSCs and enhance pigmentation outcomes [18, 19].

Another key determinant of repigmentation efficacy was the duration of gray hair, with shorter durations (< 10 years) associated with significantly better outcomes (*p* = 0.0363). This finding supports previous hypotheses that the likelihood of repigmentation diminishes over time due to progressive MeSCs depletion and irreversible follicular miniaturization [20, 21]. Furthermore, baseline graying severity influenced treatment response: moderate graying (Stage 2, 26%–50%) was associated with the most favorable outcomes, likely due to better retention and activation potential of MeSCs [20]. In contrast, individuals with advanced graying (Stages 3–4) exhibited limited improvement, underscoring the importance of early intervention for successful outcomes [4, 22].

The delivery modality and exosome concentration may also have contributed to treatment variability. While employing various treatment modalities, this study also observed efficacy in both hair regrowth and hair repigmentation [9]. Fractional radiofrequency microneedling was the most commonly used method (40%), followed by laser‐assisted delivery and manual microneedling. These techniques likely enhance percutaneous absorption by creating microchannels or thermal injury, facilitating the transdermal transport of exosomes [23]. The dilution volume per vial ranged from 0.5 to 6.0 mL, potentially affecting exosome concentration at the target site. One patient (Case 10) received two vials in just 1.0 mL, possibly leading to a more concentrated and localized effect. These procedural factors merit further standardization in future trials [24].

Mechanistically, exosomes likely exert their pigmentary effects via paracrine signaling involving bioactive cargoes such as Wnt proteins [25], miRNAs (e.g., miR‐203 and miR‐200c) [7], and growth factors. These molecules promote MeSC proliferation, MITF activation, and melanin synthesis through key signaling cascades like Melanocyte Inducing Transcription Factor (MITF) [26] and Wnt/β‐catenin [27]. Additionally, the antioxidative and anti‐inflammatory effects of exosomes may help counteract oxidative stress [2] and chronic inflammation [8], both implicated in melanocyte dysfunction and hair graying [28]. Notably, RSCEs have been shown to enhance melanin production in B16F10 melanocytes, especially when co‐cultured with hair‐derived cells such as HDP (human dermal papilla) cells (data not published). These in vitro findings suggest that RSCEs may crosstalk with various cell types within the hair follicle niche to provide stimulatory cues that promote melanin synthesis, potentially contributing to hair pigment restoration.

Despite the promising outcomes, the study has several limitations. The sample size was small, and no control or placebo group was included. The use of subjective scoring by dermatologists could introduce assessment bias. Objective tools such as melanin index measurements, or trichoscopy‐based quantification would strengthen outcome reliability. Furthermore, the long‐term durability of repigmentation remains unknown, as follow‐up beyond the treatment phase was limited.

## 5 Limitations

This study has several important limitations. First, it employed a cross‐sectional observational design without randomization or standardized regimens. The small sample size and absence of a control or placebo group further restrict the ability to attribute observed repigmentation solely to exosome therapy. Second, there was heterogeneity in treatment approaches, including variations in the number of sessions, exosome dilution volumes, and delivery modalities (RF microneedling, laser‐assisted delivery, jet device, or topical application). These procedural differences may have introduced confounding effects, making it difficult to isolate the role of exosomes from that of the devices or their interaction. Third, the correlation analysis regarding predictors of treatment response (AGA status, shorter graying duration, moderate baseline severity) should be considered exploratory only, given the small cohort size and potential confounders. Fourth, outcome assessments were based primarily on dermatologists' clinical grading, which, while blinded, remains subjective; future studies should incorporate objective measures such as melanin index or quantitative trichoscopy. Fifth, the long‐term durability of repigmentation remains uncertain, as follow‐up was limited to a few months after the final session.

Future studies should be conducted with standardized trial oversight. Regulatory classifications of exosome‐based products differ across countries, highlighting the need for harmonized guidelines and standardized clinical trial protocols. This study provides preliminary groundwork, but larger‐scale trials are required to standardize exosome source, dosage, delivery method, and frequency, as well as to identify molecular predictors of response. Long‐term follow‐up is also necessary to assess the durability of repigmentation and the need for maintenance treatments.

## 6 Conclusion

This cross‐sectional observational study provides preliminary clinical evidence supporting the efficacy of exosome‐based therapy in inducing hair repigmentation in individuals with gray hair, utilizing various treatment techniques. The majority of patients exhibited visible improvement, with treatment response significantly associated with the presence of AGA, shorter duration of graying, and moderate baseline severity. The therapy was well tolerated, with no adverse effects reported. Despite these promising results, the small sample size, absence of a control group, and short follow‐up period limit the generalizability of the findings. Larger controlled studies with standardized protocols and objective assessments are needed to confirm efficacy and optimize treatment.

## Author Contributions

S.L. contributed to study design, data collection, analysis, manuscript preparation, and clinical input. P.P.‐H.H., W.‐Y.C., E.T., and D.L. contributed case data and clinical input. All authors have read and approved the final manuscript.

## Ethics Statement

This study was conducted in accordance with the principles outlined in the Declaration of Helsinki and Good Clinical Practice guidelines.

## Consent

Written informed consent was obtained from all patients, including consent for the use of clinical information and images.

## Conflicts of Interest

The authors declare no conflicts of interest.

## Data Availability

The data that support the findings of this study are available on request from the corresponding author. The data are not publicly available due to privacy or ethical restrictions.
